# Mast cell leukemia morphologic illustration of a rare entity

**DOI:** 10.1002/jha2.571

**Published:** 2022-09-09

**Authors:** Chauncey R. Syposs, Andrew G. Evans, John M. Bennett, Jane L. Liesveld, Siba El Hussein

**Affiliations:** ^1^ Department of Pathology University of Rochester Medical Center Rochester New York USA; ^2^ Department of Medicine and The James P. Wilmot Cancer Center University of Rochester Medical Center Rochester New York USA

**Keywords:** acute basophilic leukemia, leukemia, mast cell leukemia, myelomastocytic leukemia, systemic mastocytosis

1

A 72‐year‐old woman with no oncologic past medical history presented to the emergency department with several weeks of progressive fatigue, dyspnea, night sweats, intermittent rash, and a 30 lb unintended weight loss. Initial imaging demonstrated splenomegaly (up to 22.6 cm) and attenuated bone marrow lesions, suspicious for bone marrow involvement by an occult malignancy. Her complete blood count was significant for mild anemia (hemoglobin 10.0 g/dl), thrombocytopenia (platelets 103 × 10^9^/L), with a normal white blood cell count of 10.0 × 10^9^/L. Serum tryptase and histamine levels were markedly elevated, >400 ug/L (normal range ≤ 10.9 ug/L) and 51,930 nmol/L (normal range 180–1800 nmol/L), respectively. Peripheral blood smear review demonstrated 20% immature cells, medium‐to‐large in size, with abundant cytoplasm, prominent metachromatic granules, round nuclei and immature chromatin (Figure 1A). Flow cytometry analysis of the blood revealed an aberrant mast cell population expressing CD2 (40%), CD13, CD25, CD33, and CD117; while negative for the expression of CD34, HLA‐DR, cytoplasmic MPO, and other lymphoid and monocytic markers. A bone marrow biopsy demonstrated extensive involvement by leukemic mast cells (Figure 1B–D), with characteristically round rather than spindle‐shaped nuclear contours, abundant cytoplasm, some granulated and vacuolated, and immature chromatin, with frequent mitotic figures (Figure 1D). These cells were diffusely positive for CD25, CD68, and CD117, and predominantly negative for CD30 expression, with a small subset demonstrating positivity for CD2 and tryptase (Figure 2). *KIT* mutation analysis by next generation sequencing identified *KIT* p.D816V mutation. The patient was diagnosed with mast cell leukemia (MCL). After initial diagnosis she received five cycles of Avapritinib with good response. Her serum tryptase dropped from >400 ug/L to 21 ug/L. However, her post‐treatment course was complicated by lower gastrointestinal bleeding, persistent splenomegaly and worsening anemia, and the patient passed away shortly after.

**FIGURE 1:**
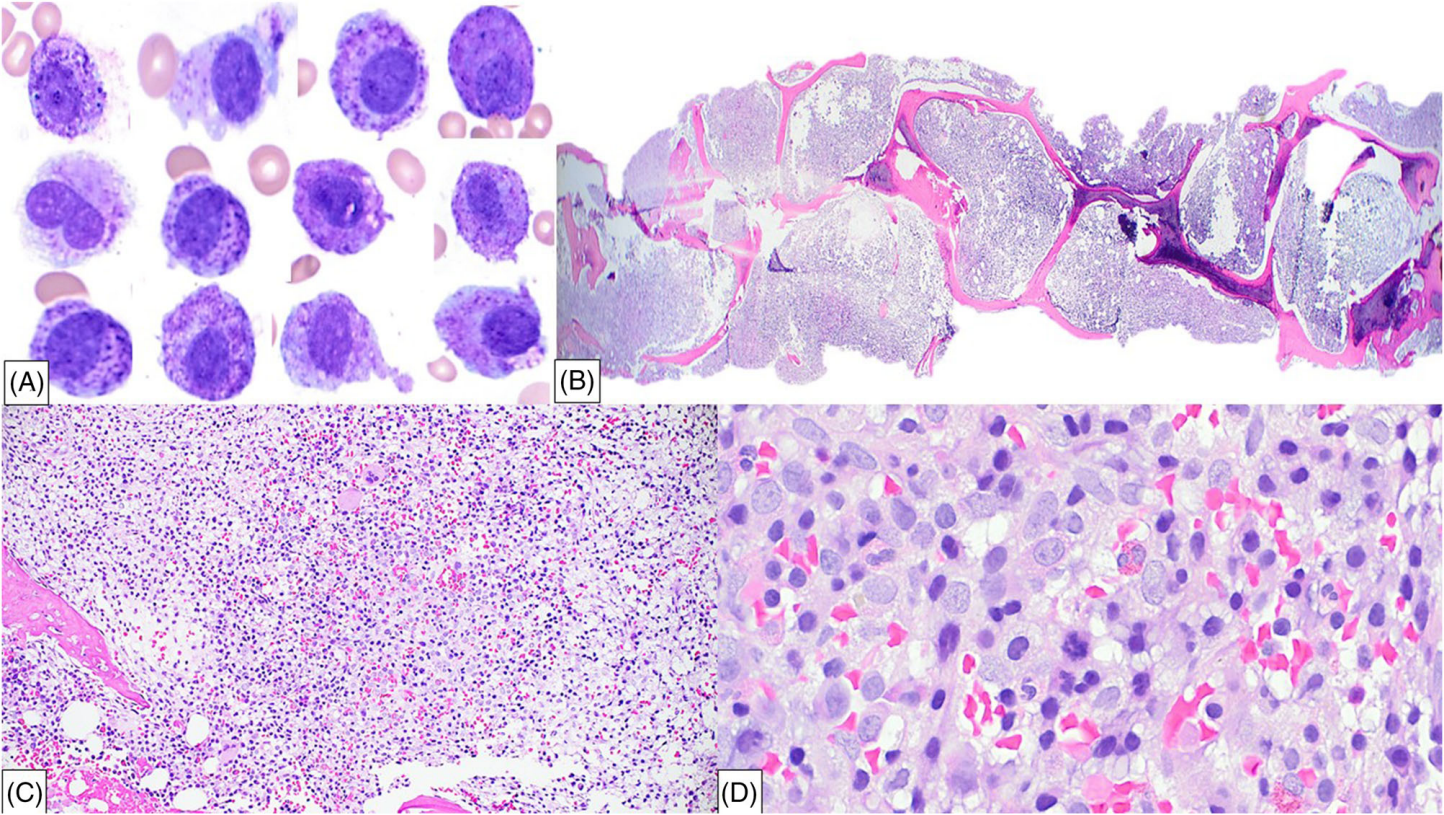
Morphologic features of mast cell leukemia: (A) Peripheral blood smear showing numerous medium to large immature mast cells with prominent metachromatic cytoplamsic granules obscuring the nuclei in few cells, round‐shaped nuclear contours with few bilobated forms, and immature chromatin. (B and C) Bone marrow biopsy almost completely replaced by leukemic mast cells, (D) with characteristically round rather than spindle‐shaped nuclear contours, abundant cytoplasm, some ganulated, and vacuolated, and immature chromatin, with frequent mitotic figures.

**FIGURE 2:**
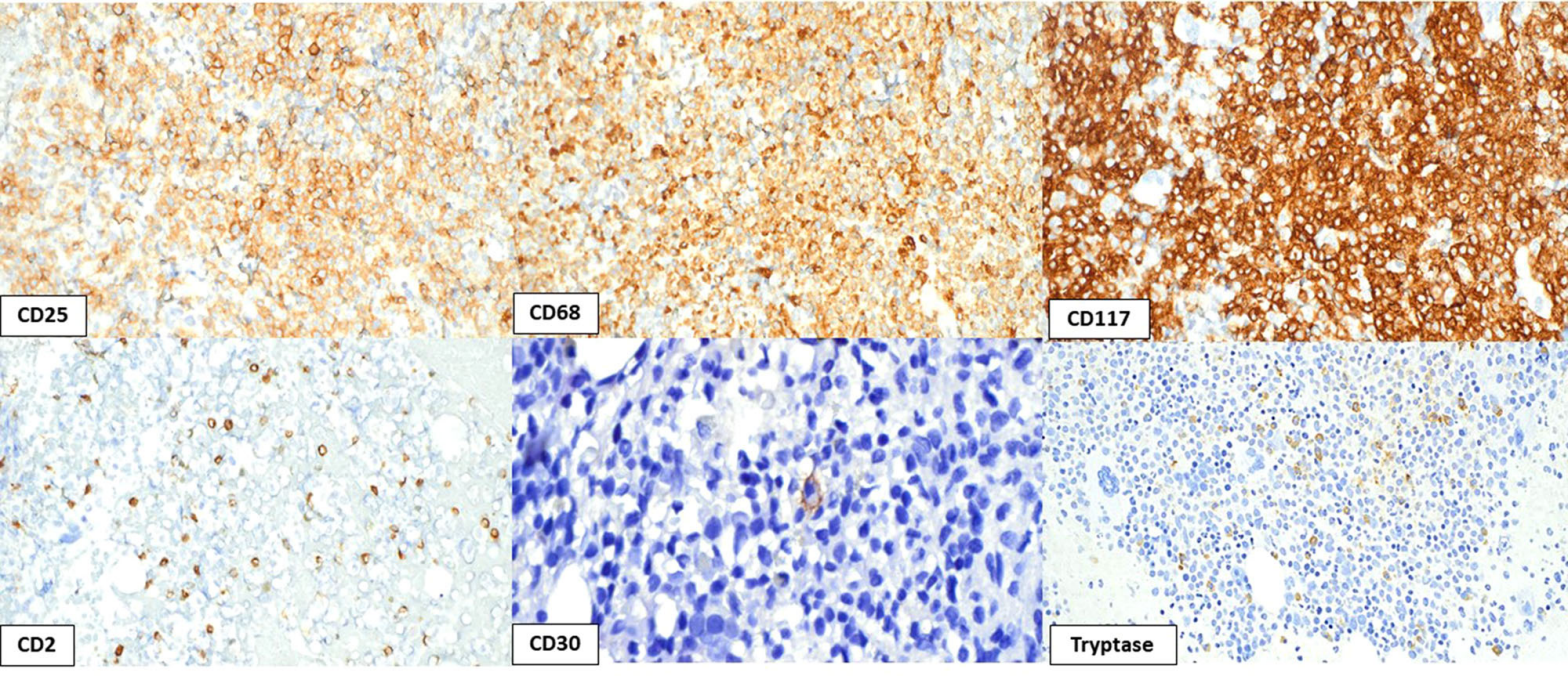
Immunophenotypic features of mast cell leukemia: An illustrative case showing positivity for markers tpically expressed by systemic mastocytosis, such as CD25, CD68, and CD117, with mostly negativity for CD30 expression, and small subset positivity for CD2 and Tryptase, highlighting the importance of performing a complete immunophenotypic panel in similar cases to highlight the mast cell derivation of the underlying process

MCL is defined as a leukemic subtype of systemic mastocytosis (SM) when mast cells are ≥20% in bone marrow aspirate smears. Unlike SM, these cells are immature with round rather than spindle‐shaped nuclei [1, 2]. A large subset of patients with MCL demonstrates a less overt (or absent) leukemic spread into peripheral blood. In this setting, the disease is termed “aleukemic” MCL (mast cells comprise <10% of all circulating blood leukocytes). MCL is further subdivided into primary MCL (absence of prior SM) and secondary MCL (progression following a previously established SM). Furthermore, MCL can be subdivided into acute MCL and chronic MCL when C‐findings (i.e., organ involvement with dysfunction, requiring cytoreduction) are present and absent, respectively. Patients with chronic MCL may respond to KIT‐targeting drugs and have a better prognosis in comparison to acute MCL. Nevertheless, over time, many patients may progress to acute MCL. MCL typically shows positivity for at least some SM‐markers, such as CD2, CD25, CD30, CD117, and Tryptase expression, highlighting the importance of performing a complete immunophenotypic panel in similar cases to highlight mast cell derivation of the underlying immature process. In addition, MCL harbors *KIT* D816V mutation in 50%–70% of cases. However, in the remainder cases, MCL may harbor atypical *KIT* mutations such as non‐D816V codon 816 mutations or non‐codon 816 mutations. Therefore, if a case of MCL is negative for *KIT* D816V mutation, *KIT* sequencing is recommended. Patients with MCL may also accumulate mutations in other genes, such as *TET2*, *SRSF2*, and *CBL*, as part of disease biology, without necessarily indicating an associated underlying hematologic neoplasm. The differential diagnosis of MCL includes mainly myelomastocytic leukemia (MML) and acute basophilic leukemia (ABL) [3]. MML is an extremely rare form of mast cell differentiation occurring in an advanced myeloid neoplasm, characterized by >10%–19% immature mast cells/ metachromatic blasts in the bone marrow and >5% myeloblasts in the bone marrow and/or peripheral blood; MML demonstrates wild‐type *KIT* and CD25 negativity, which distinguishes it from MCL. ABL is characterized by circulating immature cells with basophilic cytoplasm and coarse basophilic granules. Unlike MCL, ABL is negative for CD117 expression. However, it is positive for some myeloid markers and CD123 expression but negative for other monocytic markers. Immunophenotypic detection of abnormal cells expressing tryptase, CD25 and CD117 is helpful in distinguishing MCL from ABL.

## CONFLICT OF INTEREST

The authors declare that there is no conflict of interest that could be perceived as prejudicing the impartiality of the research reported.

## FUNDING STATEMENT

The authors received no specific funding for this work.
